# Patterns of natural selection acting on the mitochondrial genome of a locally adapted fish species

**DOI:** 10.1186/s12711-015-0138-0

**Published:** 2015-07-03

**Authors:** Sofia Consuegra, Elgan John, Eric Verspoor, Carlos Garcia de Leaniz

**Affiliations:** Department of BioSciences, Swansea University, Swansea, SA2 8PP UK; Inverness College, University of Highlands and Islands, Inverness, IV1 1SA Scotland UK

## Abstract

**Background:**

Mitochondrial DNA (mtDNA) is frequently used in population genetic studies and is usually considered as a neutral marker. However, given the functional importance of the proteins encoded by the mitochondrial genome, and the prominent role of mitochondria in cellular energy production, the assumption of neutrality is increasingly being questioned.

**Results:**

We tested for evidence of selection on the mitochondrial genome of the Atlantic salmon, which is a locally adapted and widely farmed species and is distributed across a large latitudinal cline. We analysed 20 independent regions of the salmon mtDNA that represented nine genes (*ND1*, *ND2*, *ND3*, *COX1*, *COX2*, *ATP6*, *ND4*, *ND5*, and *CYTB*). These 20 mtDNA regions were sequenced using a 454 approach from samples collected across the entire European range of this species. We found evidence of positive selection at the *ND1*, *ND3* and *ND4* genes, which is supported by at least two different codon-based methods and also by differences in the chemical properties of the amino acids involved. The geographical distribution of some of the mutations indicated to be under selection was not random, and some mutations were private to artic populations. We discuss the possibility that selection acting on the Atlantic salmon mtDNA genome might be related to the need for increased metabolic efficiency at low temperatures.

**Conclusions:**

The analysis of sequences representing nine mitochondrial genes that are involved in the OXPHOS pathway revealed signatures of positive selection in the mitochondrial genome of the Atlantic salmon. The properties of the amino acids involved suggest that some of the mutations that were identified to be under positive selection might have functional implications, possibly in relation to metabolic efficiency. Experimental evidence, and better understanding of regional phylogeographic structuring, are needed to clarify the potential role of selection acting on the mitochondrial genome of Atlantic salmon and other locally adapted fishes.

**Electronic supplementary material:**

The online version of this article (doi:10.1186/s12711-015-0138-0) contains supplementary material, which is available to authorized users.

## Introduction

Mitochondrial DNA (mtDNA) is widely used as a marker in animal population genetics, to analyse population structure and reconstruct phylogenetic relationships [1]. As a marker, some of the unique characteristics of mtDNA compared to nuclear DNA are a high rate of nucleotide substitution, maternal inheritance, and little or no recombination [2, 3]. In addition, mtDNA is generally assumed to evolve under neutral or nearly-neutral selection [4]. However, given the functional importance of some of the peptides encoded by mitochondrial genes, there is increasing interest in understanding the processes that promote and maintain mtDNA variation, including selection [5]. In contrast to nuclear DNA variation, mtDNA variation does not seem to reflect population size or ecological factors [6]. Mitochondria are responsible for 95 % of the eukaryotic cell’s energy through oxidative phosphorylation of ADP (adenosine diphosphate) to form ATP (adenosine triphosphate). Thus, some variation in mtDNA may have important fitness implications, and selection on mitochondrial genes may be influenced by environmental conditions affecting metabolic processes and may be taxon-specific [7]. Several proteins that contain mtDNA-encoded peptides participate not only in the translocation of protons and electrons but also in the regulation of mitochondrial respiration [8, 9]. The association between mtDNA- and nuclear-encoded proteins in these pathways suggests that natural selection has favoured co-adaptation to maintain optimal metabolic function [10, 11].

Variation in mtDNA is related to a range of environmental conditions, including anoxic subterranean environments in *Caviomorph* rodents [12], altitude in monkeys [13], and latitudinal clines in killer whales (*Orcinus orca*) [14] and Pacific salmon (*Oncorhynchus sp.*) [15], which may be suggestive of an adaptive function. A possible role of selection in mtDNA evolution is further supported by evidence from studies on mitochondrial dysfunction in human pathologies [16–18], with more than 250 pathogenic mtDNA mutations identified [19] and by the observed regional distribution of human haplotypes [20, 21]. The effects of strong purifying selection on mammalian mtDNA have been documented by comparing the inheritance of patterns of mutation in mouse lines that have a high frequency of mtDNA mutations with those in the human mtDNA [22]. In addition, the lack of correlation between population size and mtDNA genetic diversity can be attributed to deviations from the neutral expectation [23] and patterns of non-neutral mtDNA polymorphisms can be explained, at least in part, by negative frequency-dependent selection, whereby the relative fitness of a particular haplotype decreases as its frequency increases in the population [24]. However, the role of positive selection and co-adaptation between nuclear and mtDNA in the evolution of mtDNA remains controversial [6, 7, 25]. Given the popularity of mtDNA in population genetics studies, understanding the selective mechanisms that affect its evolution is important for interpreting studies on the ecology and evolution of natural populations.

Salmonids are good models to study mtDNA evolution because their current geographical distribution is, in many cases, the result of recolonisation from a few refugia since the last glaciation period [26–28]. This enables geographical comparisons between recently diverged populations. In addition, due to the economic importance of farmed salmonids, there is a wealth of data on mtDNA variation in this species, which has been used for phylogenetic reconstruction of salmonids [29–33], and there is evidence that shows that mtDNA variation is linked to environmental conditions [34] and development rates [35]. Within the salmonids, Atlantic salmon (*S. salar*) is arguably the species that has suffered the greatest decline in abundance [36], mainly due to over-exploitation, habitat loss, interactions with escaped farm fish [37], and also possibly climate change [38]. The typically anadromous life cycle and homing behaviour of Atlantic salmon make them particularly suited for the study of local adaptations [39]. Variation in the distribution of salmon mtDNA haplotypes has been observed across the range of the species [40] and at different geographical scales [41, 42]. Hence, mtDNA haplotype frequencies differ markedly, not only between Baltic and Atlantic populations [26, 31], but also between anadromous and non-anadromous sympatric populations [43], and even between adults that return to spawn in the same river but at different times of the year [44]. Yet the functional significance of mtDNA variation in this species remains largely unknown. Previous work on allozyme variation in Atlantic salmon strongly suggested that the nuclear-encoded mitochondrial malic enzyme MEP-2*, is under selection. Polymorphisms in the MEP-2* gene are associated with temperature, growth, and age at maturation along a latitudinal cline and also with mitochondrial oxygen consumption [44–47]. Studies on salmonid hybrids have shown that thermal adaptation varies greatly between hybrids depending on maternal origin, which suggests that salmonid mitochondria respond differently to temperature variations [5].

Much of the observed variation in the life history of anadromous Atlantic salmon populations appears to be determined by the rate of growth achieved early in life, which suggests that maternal effects may play an important role in this species [48, 49]. Thus, whether male salmon mature in freshwater before migrating to the sea (‘precocious parr’), or after spending one to three winters at sea (sea-run) may depend on size and/or growth thresholds attained as juveniles [50, 51], which, in turn, depend on size at hatching, and ultimately egg size [52, 53]. Growth rate in salmonids is mainly regulated by adjusting activity, feeding rate, and metabolic rate [54]. Variation in the metabolic rate of salmonids early in life has low heritability but is strongly influenced by maternal effects [54, 55], which can have long-term consequences for life history strategies and fitness-related traits such as growth and maturation [53, 54]. Hence, given the putative role of the mitochondrial genome on fish growth and the extent of the genetic variation that exists for growth-related traits in Atlantic salmon [39, 56], analysis of its mitochondrial genome may provide clues about the role of natural selection on the evolution of the mitochondrial genome in vertebrates.

In this study, we tested the hypothesis that mtDNA variation in Atlantic salmon is shaped by natural selection. To this end, we analysed the sequences of nine mitochondrial genes that are involved in the physiological regulation of oxidative phosphorylation (OXPHOS pathway) of ADP into ATP and of the D-loop control region in 29 salmon populations that cover the European range of this species. Evidence for selection was sought for by comparing synonymous and non-synonymous nucleotide substitution rates and by examining the chemo-physical changes that non-synonymous substitutions were predicted to have on the structure of several proteins for populations that are distributed across an extensive latitudinal cline.

## Methods

### Origin of samples and DNA extraction

**Fig. 1:**
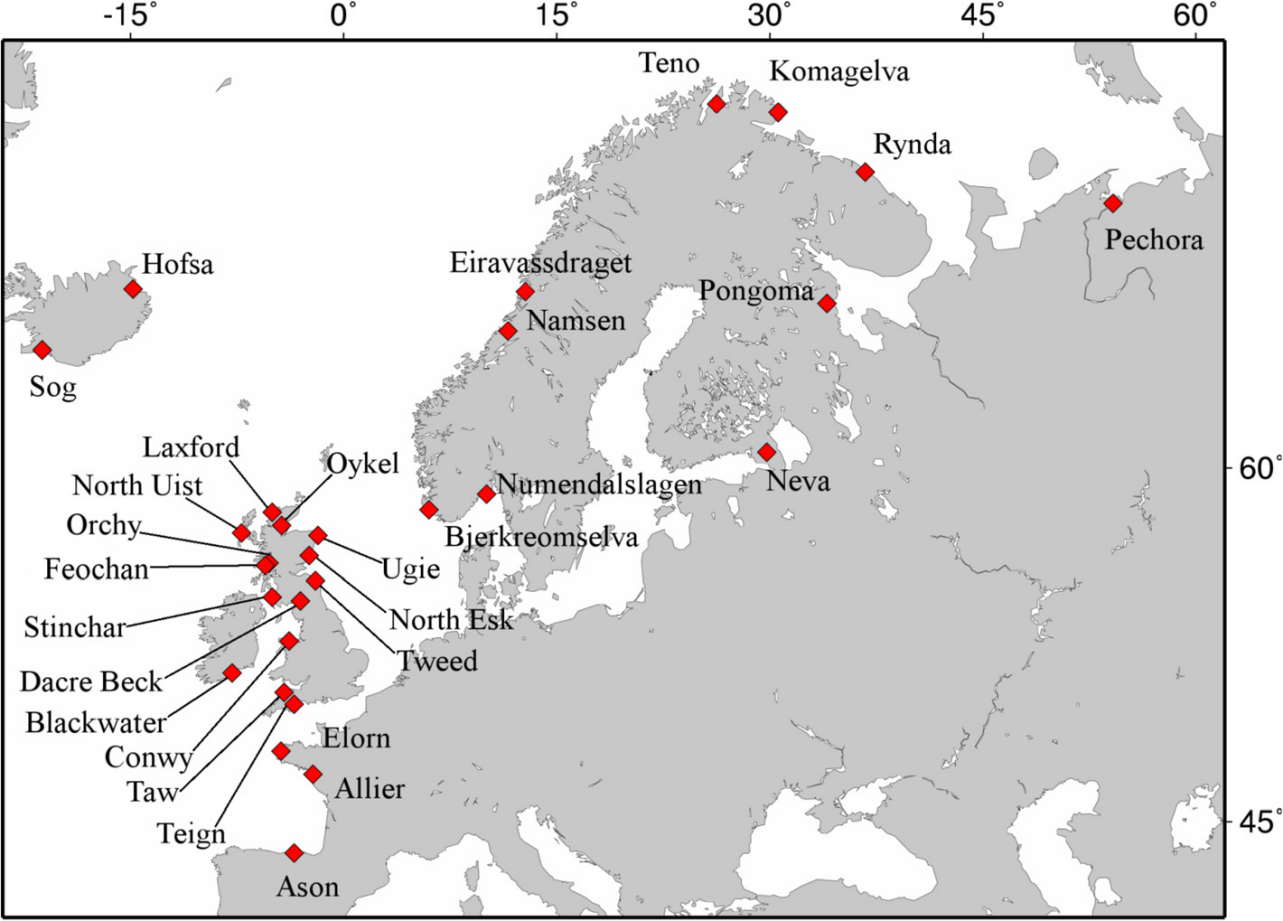
Location of the populations studied

A total of 338 Atlantic salmon samples were collected from 29 rivers that represented the entire range of the species in Europe (Fig. 1). Samples consisted of fin clips from 6 to 12 individuals per river, which have been collected over the last two decades and stored in ethanol (see Additional file 1: Table S1), as detailed in Fridjonsson et al. [57]. DNA was extracted using the Qiagen DNeasy blood and tissue kit (Qiagen) according to the manufacturer’s instructions. Twenty independent salmon mtDNA regions that were between 311 and 384 bp long and represented partial sequences of nine mtDNA genes (*ND1*, *ND2*, *ND3*, *COX1*, *COX2*, *ATP6*, *ND4*, *ND5*, and *CYTB*; [40]) were amplified and sequenced for each individual using a 454 Titanium FLX sequencer (Roche, 454 Life Sciences) [57]. Overall, 7215 bases of mtDNA were sequenced per individual (Table 1).

**Table 1:** Details on the mtDNA amplicons sequenced from Atlantic salmon samples collected across the European species’ range

Amplicon	BP	Genes	Start	End
1	380	*Dloop*	654	1033
2	78	*ND1*	3770	3847
3	371	*ND1*	3872	4242
4	368	*ND1*	4269	4636
5	324	*ND1/tRNA-Ile/tRNA-Gln/tRNA-Met*	4657	4980
6	361	*ND2*	5133	5493
7	343	*ND2*	5512	5854
8	371	*COX1*	6960	7330
9	385	*COX1*	7363	7747
10	358	*COX2*	8218	8575
11	371	*COX2/tRNA-Lys*	8579	8949
12	316	*ATP6*	9317	9632
13	357	*ND3/tRNA-Gly*	10 645	11 001
14	412	*ND4/ND4L*	11 116	11 527
15	362	*ND4*	11 551	11 912
16	369	*ND4*	11 935	12 303
17	345	*ND5*	14 331	14 675
18	371	*ND5/ND6*	14 703	15 073
19	364	*CYTB*	15 396	15 759
20	352	*CYTB*	15 785	16 136

### Selection analyses

Partial genome sequences were aligned against the *S. salar* reference sequence (NC_001960.1; [See Additional file 1: Table S2]) using MEGA 5 [58] as in [57]. A phylogenetic tree for the selection analyses was constructed based on the assembled partial mtDNA genome sequences using the PHYLIP software version 3.69 [59] and it was bootstrapped (1000 repeats) with SEQBOOT. A consensus tree was built using CONSENSE (see Additional file 2: Figure S1).

First, non-coding regions of the genome and stop codons were removed to conduct a whole-genome scan to detect evidence of selection. Then, the remaining data (including 174 codon substitutions) were analysed using two codon-based methods implemented in the HYPHY v0.99+ beta package [60]: an approximate hierarchical Bayesian method i.e. FUBAR (Fast, Unconstrained Bayesian AppRoximation) [61] and a mixed effects model, i.e. MEME (Mixed Effects Model of Evolution) [61]. MEME is based on a mixed effects model that allows the rate of nonsynonymous (dN) and synonymous (dS) substitutions (dN/dS) to vary from site to site (fixed effect) but also from branch to branch at a site (random effect). Results from MEME are consistent with those obtained with other methods that detect pervasive selection (such as REL and FEL) but are generally more powerful for the detection of episodic selection [61]. FUBAR is considered to be a more robust approach than random effects models since it uses a highly flexible and less restrictive setting and thus is less sensitive to model specification, particularly when the strength of selection varies across different sites [62]. For the analyses, we used the best-fitting nucleotide substitution model that was identified by HyPhy for each gene i.e. TrN for *ND1*, HKY85 for *ND2*, F81 for *ND3*, TrN for *ND4*, HKY85 for *ND5*, F81 for *ATP6*, HKY85 for *COX1*, F81 for *COX2*, and (010000) for *CYTB*. Codon models incorporate information from nucleotide and amino acid compositions to estimate the rate at which amino acids are replaced (dN) versus the rate at which they are preserved (dS). Fitting the best model by taking into account that different residues are exchanged at different frequencies, increases the accuracy of detection of natural selection based on sequence alignments [63]. Synonymous and non-synonymous substitution rates per population are in Table S3 (see Additional file 1: Table S3). For each method, we applied the following thresholds P < 0.1 for MEME and P > 0.9 for FUBAR.

The presence of directional selection (positive) was also analysed by comparing destabilizing amino acid changes using the software TreeSAAP that measures the selective influences on 31 structural and biochemical amino acid properties [64]. This method compares the distribution of observed changes in the DNA sequences with the predicted distribution of random changes under neutrality. TreeSAAP analyses were based on the phylogenetic tree described above with the ancestral state estimated with baseml in PAML v4.2 [65]. A z-test was used to evaluate the effects of amino acid changes, which are categorized into eight magnitude categories, category 1 being the most conservative in terms of physiochemical change and category 8 being the most radical. In our analyses, we considered only magnitude categories 6 to 8 for which amino acid changes had the strongest statistical support (*P <* 0.001) [64, 66], and only amino acid sites, that were supported by at least two independent methods, were considered to be under positive selection.

### Spatial distribution of mtDNA codon substitutions under selection

We used the spatial clustering program GENELAND [67, 68] to estimate the number of panmictic groups and locate the spatial boundaries between them. The spatial distribution of these groups was visualized with an unbiased linear kriging smoother using Systat 10.0. ‘Kriging’ is an interpolation technique that is commonly applied in geostatistics and uses local information around sampling points to extrapolate complex and irregular spatial patterns. It produces the best linear unbiased estimation of a stochastic process by generalized least-squares, fitting a surface to irregularly spaced, spatially autocorrelated data [69]. Previously, we used this technique to reconstruct the phylogeography of Atlantic salmon mtDNA haplotypes [27]. We used generalized linear models in R 3.1.3 with a binomial or quasibinomial link to examine variation in the proportion of fish with amino acid substitutions under selection with latitude, longitude, and summer and winter temperatures (the latter obtained from [70]).

## Results

Among the 174 codon substitutions tested, several were identified as being under selection by different methods, in particular, three substitutions caused by a base pair change in each of the genes *ND1*, *ND3* and *ND4*, which encode mitochondrial NADH dehydrogenase subunits. These changes occurred at nucleotide positions: 11937:A/G in *ND4*, identified under positive selection by MEME, FUBAR and TreeSAAP and 4578:G/A and 10963:C/A, in *ND1* and *ND3* respectively, identified under positive selection by FUBAR and TreeSAAP. Thirty-three additional sites were identified by FUBAR as being under purifying selection (Table 2). An error rate of 0.006 was estimated by FUBAR over all the sites and a q value of 1 was estimated by MEME for all genes, which is a very conservative estimate based on Sime’s false detection [71].

**Table 2:** Results obtained from tests of selection on the mitochondrial genome of the Atlantic salmon

Locus	Position	Type of selection	MEME		FUBAR		TreeSAAP (Category)
			ω^+^	*P*	dN-dS	Post. *P*	
*ND1*	3878:C/T	-			−9.924	0.997	
	3926:C/T	-			−5.068	0.920	
	3989:A/G	-			−8.576	0.995	
	4424:C/T	-			−4.683	0.913	
	4517:G/A	-			−4.613	0.993	
	4568:C/T	-			−4.683	0.918	
	*4578:G/A*	*+*	*>100*	*0.175*	*3.793*	*0.918*	Alpha-helical tendencies (6)
	4718:C/T	-			−5.153	0.902	
*ND2*	5161:A/G	-			−5.017	0.923	
	5290:A/G	-			−4.086	0.914	
	5341:T/C	+	>100	0.564	−0.328	0.569	Alpha-helical tendencies (6)
	5365:C/T	-			−7.495	0.991	
	5416:A/G	-			−6.931	0.987	
	5768:G/A	+	>100	0.216	0.855	0.805	Alpha-helical tendencies (6)
	5812:C/A	-			−3.339	0.902	
*COX1*	6970:C/T	-			−6.446	0.987	
	7021:A/G	-			−4.431	0.906	
	7039:A/G	-			−4.428	0.905	
	7483:G/A	-			−7.016	0.997	
	7684:T/C	-			−6.419	0.983	
*COX2*	8492:T/C	-			−4.749	0.888	
*ATP6*	9281:A/G	-			−3.654	0.902	
	9314:T/C	-			−2.981	0.891	
	9356:C/T	-			−2.984	0.893	
	9551:A/G	-			−1.253	0.928	
*ND3*	*10963:C/A*	*+*	*>100*	*0.423*	*2.706*	*0.901*	*Equil const ion COOH (8)*
	10879:T/C	-			−6.959	0.996	
*ND4*	11489:A/G	-			−3.031	0.979	
	11693:C/T	-			−9.421	0.989	
	11762:T/C	-			−8.488	0.994	
	11891:G/A	-			−1.439	0.915	
	11906:C/T	-			−4.551	0.912	
	***11937:A/G***	***+***	***>100***	***0.006***	***7.564***	***0.972***	***Power to be at C-terminal (6)***
	12167:A/G	-			−6.616	0.982	
*ND5*	14405:T/C	-			−4.071	0.900	
	14570:C/T	-			−4.718	0.923	
*CYTB*	16178:A/G	-			−9.805	0.996	
	16361:G/A	-			−2.654	0.950	

Type of selection: positive (+) or negative (−). Sites in bold correspond to those identified by the three different methods as being under selection, those in italics are sites identified by two different methods. Thresholds for each method: MEME P < 0.1, FUBAR p > 0.9 & amino acid change categories 6–8

TreeSAAP detected five significant changes in amino acid properties (Table 2). These included the change at position 4578:G/A in *ND1*, which increased the alpha-helical tendency to a magnitude category of 6 (*P* < 0.001) as a result of a change in amino acid hydrophobicity and the change at position 11937:A/G in *ND4*, which increased the power to be at the C-terminal of an alpha helix to a magnitude category of 6. In addition, the change at position 10963:C/A in *ND3* increased the equilibrium constant (ionization of COOH) to a magnitude category of 8, and the change at position 11592:G/A in *ND4* increased the total non-bonded energy to the most radical magnitude category (8). Other radical changes in amino acid properties were identified in *COX1* and *COX2* genes although the z-values suggested that they were not under directional selection. The mutation at position 8402:T/C in *COX2* that causes an amino acid change was present in all individuals that also carried the mutation at position 5768: G/A in *ND2*, which was identified as being under selection by TreeSAAP; these mutations were private to two arctic populations i.e.in the rivers Teno and Rynda (Fig. 1). Similarly, the polymorphism 9469:G/A in *ATP6* was private to a single population i.e. in the Pechora river (Fig. 1), which is also located within the arctic range and was found to be under positive selection with TreeSAAP, with a radical change in magnitude category to seven for isoelectric point (*P* < 0.001), but not in any other test. Linkage disequilibrium (LD) analyses identified 8 % of the comparisons (1042/11628) with significant LD between variable sites after Bonferroni correction but none of them involved the three sites identified as being under positive selection by two or three methods (the only sites we considered).

Variation in the frequencies of the ND1 substitution (range = 0.00 to 0.17, n = 6 rivers involving eight individuals) and the ND3 substitution (range = 0.00 to 0.17, n = 3 rivers involving three individuals) were too small to allow for meaningful statistical modelling. Variation in frequencies of the ND4 substitution among rivers was negatively correlated to latitude (parameter estimate = −0.12, SE = 0.05, *P* = 0.02) and summer temperature (estimate = −0.027, SE = 0.13, *P* = 0.057), and positively correlated to longitude (estimate = 0.04, SE = 0.01, *P* = 0.006) using a quasibinomial link to correct for overdispersion (residual deviance = 60.580 on 25 degrees of freedom). Although predictors were correlated, all variance inflation factors (vif) were less than 5 (vif summer temperature = 2.57; vif latitude = 3.76; vif longitude = 2.13) which indicated that multicolinearity was probably not the problem.

**Fig. 2:**
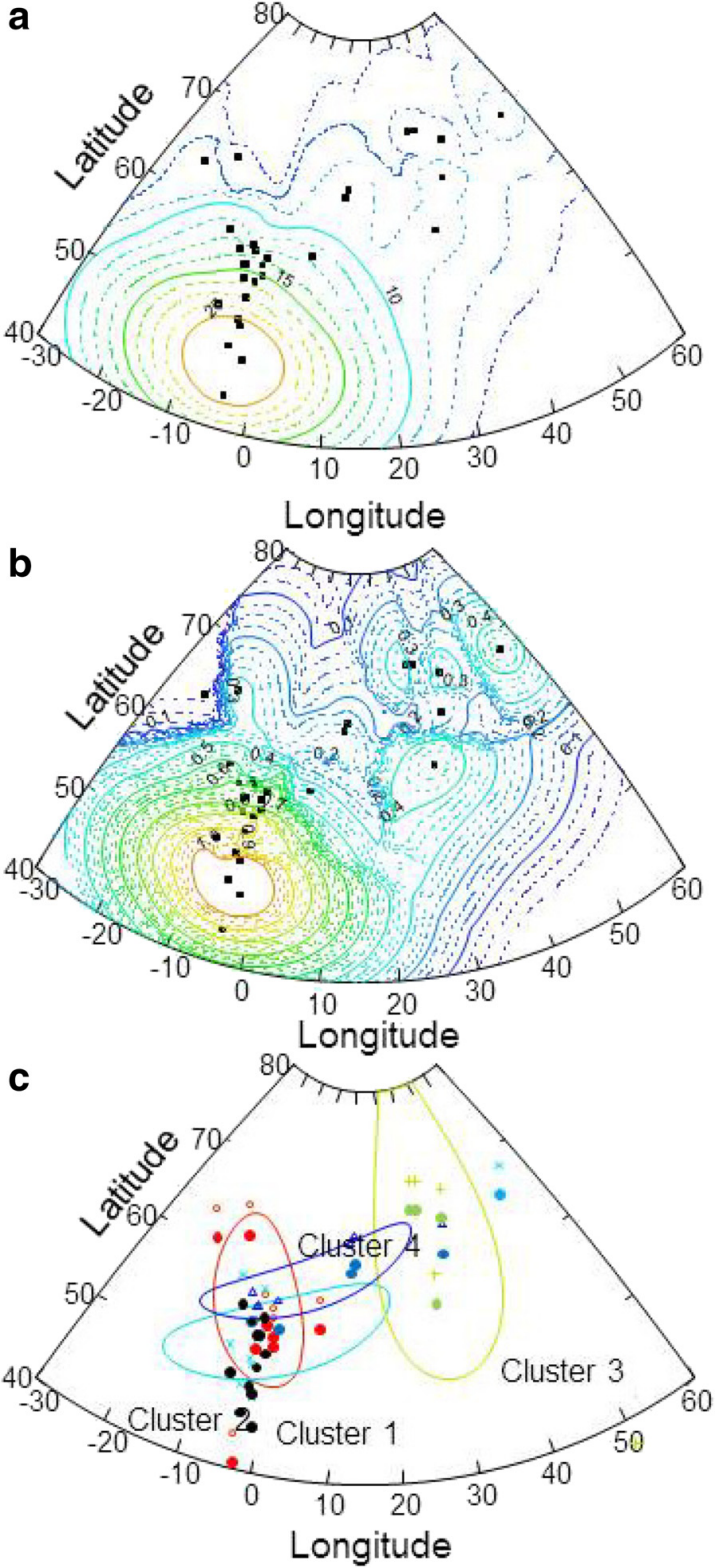
Spatial distributions. **a** average summer temperatures. **b** Atlantic salmon mitochondrial *ND4* 11937:A/G mutation under positive selection visualized by krigging smoothing. **c** homogeneous *ND4* 11937:A/G mutation clusters detected by GENELAND

Analysis of *ND4* haplotype frequencies by GENELAND rejected the null hypothesis of a single panmictic population, and, instead, it identified four homogenous clusters that were separated according to location and thus, also likely according to temperature profile, as indicated by GLM modelling (Fig. 2).

## Discussion

Sequence analysis of nine genes involved in the OXPHOS pathway revealed signatures of both purifying and positive (directional) selection in the mitochondrial genome of the Atlantic salmon, a non-model organism that has been widely used in studies of local adaptation [39]. Although caution must be exercised given the linked nature of mitochondrial single nucleotide polymorphisms (SNPs), the relatively small sample size and the possibility of founder effects in small populations, we are confident that our study provides strong support for a putative role of selection in shaping mtDNA variability in Atlantic salmon. We used two methods of DNA sequence analysis, along with inferences that were drawn directly from the chemical properties of the amino acids involved and from allele frequencies, and excluded evidence for selection based on only one method.

One of the sites on the *ND4* gene was identified as being under positive selection by three methods and two other sites on the *ND1* and *ND3* genes were identified by two complementary methods. The proteins encoded by these three genes are part of the NADH dehydrogenase complex 1, a L-shaped protein complex that consists of at least 35 subunits [72]. NADH dehydrogenase is the first enzyme complex of the mitochondrial electron transport system (ETS) that transfers electrons from NADH to O_2_. Although there is currently no crystal structure available for the proteins of this complex in teleost species, the proteins that are encoded by the salmon mitochondrial genes *ND2*, *ND4*, *ND5*, and *ND6* are homologous to those encoded by *NuoN*, *NuoM*, *NuoL*, and *NuoJ* in *E. coli*, for which crystal structures have recently been mapped [73]; the crystal structure of ND1 has not been mapped due to its solubility. The conservative nature of these proteins means that they can be used to predict the likely effects of mutations in salmon mtDNA on the structure and function of complex 1 by mapping amino acid changes onto the *E. coli* crystal structure.

The regions detected to be under selection were located on the trans-membrane (TM) helix TM1 in ND4 and TM6/TM7 in ND1. The TM1/ND4 helix (that includes the *ND4* mutation 11937: A/G) is part of the macromolecule that faces the lipid bi-layer and it is likely that it has a structural function. This polymorphism occurs in a region that is regionally conserved [46], which suggests that mutations in this region might have some effect on enzyme function. ND4 variation was present in all populations, except for one Icelandic population, but GENELAND and generalized linear modelling ruled out panmixia and instead revealed a correlation between the frequency of this mutation and latitude, longitude and average summer temperature.

In mammals, ND4 together with ND2 and ND5 are considered to be the actual proton pumps in complex 1. Thus, variation in these subunits may affect the efficiency of the proton-pumping process, for example through chemical changes that may alter proton translocation [74]. ND1 has a critical role in the assembly of complex 1 [75], and the region where the salmon polymorphism *ND1* 4578:G/A was identified was suggested to contain a binding site for the nDNA mitochondrial products 49 KDa and PSST in bovine mitochondria [76], PSST being an important part of the electron transport chain. ND1 itself serves as a conduit in the transfer of electrons from ubiquinone to its binding pocket in the Q-module [77]. TreeSAAP results suggest that the mutation present in salmon *ND1* changes the structure and shape of this binding site since the non-synonymous change increases the alpha-helical tendency of this region. Thus, the mutation *ND1* 4578:G/A may represent a response to changes in nDNA mitochondrial products 49KDa and/or PSST, perhaps as a result of a process of co-evolution between the mitochondrial and the nuclear-encoded subunits [78].

We did not find clear evidence for selection in *ND5* and *ND2*, two loci that could be functionally relevant and seem to have evolved under selection in Pacific salmon and other vertebrates based on codon-based evidence and changes in the amino acids [15, 74]. However, we found a significant amino acid change associated to a base change in *ND2* (5768:G/A), which is located in the TM7 in Atlantic salmon. This change was correlated with non-synonymous substitutions at positions 5752, 5812 and 5759, two of which (5768 and 5759) were in LD. TM7 is part of the conserved core of the antiporter-like ND2 protein, which has been proposed to be a proton translocation pathway adjacent to TM7 [73, 79]. Given that the polymorphic regions are found close to the entrance of the putative pathway, these non-synonymous changes might affect the efficiency of proton translocation.

Analysis of amino acid properties indicated that the base change *ND2* (5768:G/A) affects the secondary structure of the protein by increasing the alpha-helical propensity of the region and, possibly, producing a kink in the trans-membrane 292 helix, which is similar to that found in another region of TM7. In terms of spatial patterns, the *ND2* mutations occur mainly in the populations of two adjacent rivers within the Arctic Circle, which are both located at the northernmost limit of the species’ range i.e. rivers Teno and Rynda. A similar latitudinal pattern of mtDNA mutations was recently reported in Pacific salmon [15] and killer whales [14], which suggests that differential patterns of selection in the northern and southern ranges may act on mitochondrial genes.

In general, our results point to a non-random geographical pattern of mtDNA mutations associated with the most northerly salmon populations. Positive selection acting directly or indirectly on the mitochondrial genes of northern populations may reflect selection for higher aerobic capacity (and thus metabolic efficiency) at low temperatures. Increased aerobic capacity has been related to increased endothermy in tuna and bill fish (*Scombridae*) [80], thermal adaptation [14, 15, 21], and adaptation to decreasing oxygen availability [12, 13]. However, we did not find consistent evidence for selection in Atlantic salmon, and the distribution of neutral markers suggests that the pattern could be also influenced by genetic drift and founder effects [81, 82]. For example, the populations in the rivers Teno and Rynda, which are characterised by specific mutations in the mitochondrial *ND2* gene, tend to cluster together and to separate from other populations [40], which could be the result of founder effects and/or genetic drift.

Some of the observed polymorphisms under selection (e.g. *ND1*) could also be the result of co-evolution between nuclear and mitochondrial proteins due to environment-genome-genome interactions [5]. Such co-evolution has been reported between cytochrome c oxidase (a mtDNA gene product) and cytochrome c protein (a nDNA product) in primates [83], as well as between ND1/ND4 and the nuclear-encoded NDUFA1 in humans [78], although the adaptive role of mitochondrial variation in these cases is still unclear [84].

## Conclusions

In summary, our results provide evidence for both purifying and positive selection acting in several Atlantic salmon mtDNA genes that are involved in the OXPHOS pathway and for a non-random geographical pattern of mtDNA mutations. The properties of the amino acids involved suggest that at least some of the mutations identified as being under positive selection might have functional implications, most likely in relation to metabolic efficiency, as suggested for other species. However, to understand the relative importance of neutral and non-neutral forces, a better understanding of regional phylogeographic structuring in this species is required. In addition, experimental evidence is clearly needed to clarify the role of selection in shaping mtDNA variation in salmonids, as undertaken for other species [22, 85].

## Additional files

Additional file 1: Table S1. Sample size (N) and number of individuals with particular base changes per river. Sample size (N) and number of individuals per river with different base changes in the mitochondrial DNA of Atlantic salmon populations across Europe. **Table S2.** Localization of base changes in relation to reference genome NC_001960.1. Localization of the base pair changes in the mitochondrial genome of Atlantic salmon that were detected in the current study. **Table S3.** Haplotype and nucleotide diversity and number of synonymous and non-synonymous substitutions per river. Diversity in the mitochondrial DNA of Atlantic salmon in populations across Europe. Additional file 2: Figure S1. Phylogenetic tree of the partial mtDNA sequences constructed with PHYLIP using 1000 iterations. Phylogenetic tree of partial mitochondrial DNA sequences from Atlantic salmon across Europe.
