# Supplementary figures and images for: Patterns of natural selection acting on the mitochondrial genome of a locally adapted fish species
Source: Genet Sel Evol. 2015 Jul 3;47(1):58. doi: 10.1186/s12711-015-0138-0 (PMC4490732; doi:10.1186/s12711-015-0138-0)

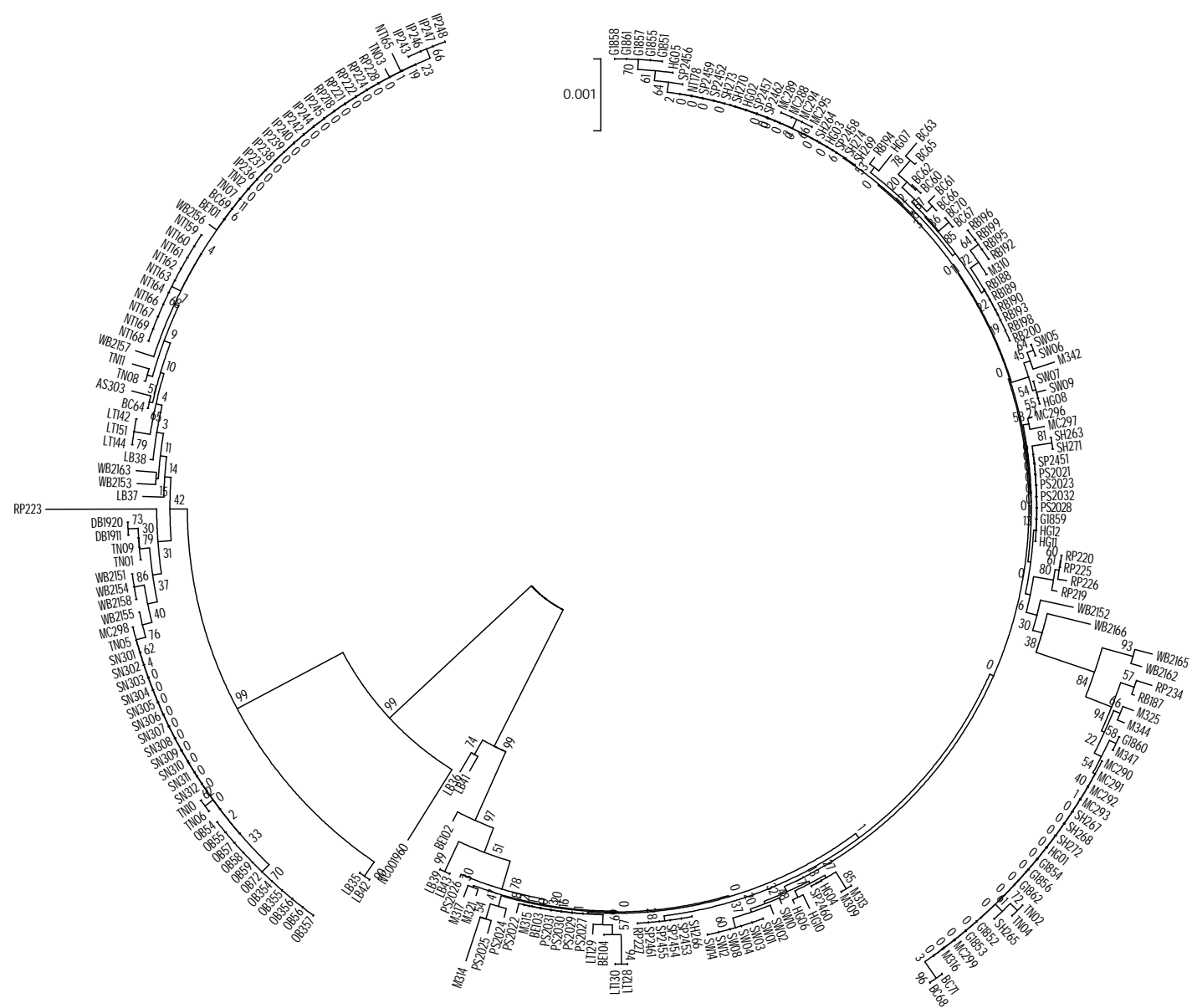

Supplement: Additional file 1: Table S1. — Sample size (N) and number of individuals with particular base changes per river. Sample size (N) and number of individuals per river with different base changes in the mitochondrial DNA of Atlantic salmon populations across Europe. Table S2. Localization of base changes in relation to reference genome NC_001960.1. Localization of the base pair changes in the mitochondrial genome of Atlantic salmon that were detected in the current study. Table S3. Haplotype and nucleotide diversity and number of synonymous and non-synonymous substitutions per river. Diversity in the mitochondrial DNA of Atlantic salmon in populations across Europe. [file 12711_2015_138_MOESM1_ESM.xlsx]
